# Comparing Early Outcomes and Complications Between Total Ankle Arthroplasty and Ankle Arthrodesis in Patients with Ankle Osteoarthritis: Big Data Analysis

**DOI:** 10.3390/jcm14092909

**Published:** 2025-04-23

**Authors:** Assil Mahamid, Lior Laver, David Maman, Amir Abu Elhija, Mohammad Haj Yahya, Daniel Haverkamp, Yaron Berkovich, Eyal Behrbalk

**Affiliations:** 1 Department of Orthopedics, Hillel Yaffe Medical Center, Hadera 3810101, Israel; 2 Rappaport Faculty of Medicine, Technion University Hospital (Israel Institute of Technology), Haifa 3200003, Israel; 3 Carmel Medical Center, Technion-Israel Institute of Technology, Haifa 2611001, Israel; 4 Department of Orthopedic Surgery, Xpert Clinics, 1101 EA Amsterdam, The Netherlands

**Keywords:** total ankle arthroplasty, ankle arthrodesis, national inpatient sample, big data

## Abstract

**Background:** End-stage ankle osteoarthritis (OA) severely limits function and quality of life. Total ankle arthroplasty (TAA) and ankle arthrodesis (AA) are key surgical interventions when conservative treatment fails. This study compares TAA and AA outcomes using a national dataset to inform patient-centered care. **Methods:** A retrospective analysis of 27,595 patients undergoing TAA or AA from 2016–2019 was conducted using the National Inpatient Sample. Propensity score matching addressed baseline differences. **Results:** Primary OA was more prevalent in TAA (85.9%) than in AA (55.4%). TAA utilization rose from 73% to 78% (*p* < 0.0001), while AA declined. TAA patients were older (65.6 vs. 59.7 years), more often Medicare-insured, and predominantly Caucasian. AA patients had higher rates of comorbidities, including diabetes, obesity, lung disease, and mental disorders (all *p* < 0.0001). **Conclusions:** TAA and AA cater to distinct patient profiles. TAA is increasingly favored and associated with lower immediate risks, though procedure choice should be individualized based on comorbidities and risk profiles. These insights support evidence-based decision-making in end-stage ankle OA management.

## 1. Introduction

Osteoarthritis (OA) is characterized by joint pain and dysfunction resulting from significant joint degeneration affecting more than 10% of individuals over the age of 60 [1]. Ankle osteoarthritis, in particular, leads to severe pain, impaired mobility, and significant disability, negatively impacting quality of life [1,2]. While most cases of ankle osteoarthritis stem from previous trauma, it can also arise from long-standing inflammatory conditions such as rheumatoid arthritis or primary OA [1,2,3,4]. End-stage osteoarthritis is identified by a combination of severe, persistent symptoms that often lead patients to consider surgical intervention [5]. Patient selection for total ankle arthroplasty (TAA) and ankle arthrodesis (AA) remains a critical determinant in achieving optimal outcomes for end-stage ankle osteoarthritis. The ideal candidate for TAA is typically characterized as a middle-aged or elderly individual with end-stage ankle arthritis refractory to conservative management. Conversely, AA is generally indicated for younger patients, those with substantial deformities, compromised bone quality, or ligamentous insufficiency [6].

Ankle arthrodesis is a well-established treatment for end-stage ankle arthritis, with several techniques offering distinct benefits and drawbacks [7]. Open ankle arthrodesis (OAA) allows direct joint visualization, making it suitable for complex deformities, but is associated with longer recovery and higher risks of wound complications and infections [8,9]. Arthroscopic ankle arthrodesis (AAA) is minimally invasive, offering shorter hospital stays, faster recovery, lower infection rates, and higher fusion rates, though it may be limited in complex cases and requires specialized expertise [8,9,10]. Internal fixation (IF) provides stable fixation with high union rates and low complication rates, but carries risks such as hardware failure and malalignment [11]. External fixation (EF), often used in infection-prone or severely deformed joints, enables gradual correction but is associated with longer operative times, increased blood loss, and higher risks of deep infections and amputations [12,13]. Each method should be selected based on patient-specific factors and surgical goals [11].

The decision-making process between TAA and arthrodesis remains complex, with a paucity of definitive guidelines. Consequently, clinicians must conduct a comprehensive evaluation of individual patient factors to determine the most appropriate intervention. Meticulous patient selection is paramount in ensuring favorable long-term outcomes for both procedures in the management of end-stage ankle osteoarthritis. AA and TAA are considered the standard operative interventions when nonoperative management has proven ineffective, and the likelihood of success with joint-preserving procedures is minimal [14,15]. AA has long been regarded as the gold standard. The primary advantage of arthrodesis is the potential restoration of walking ability through pain relief and an enhanced quality of life [16], coupled with a decreased risk of deformity [17], yet neither restores normal gait pattern. However, the resultant loss of ankle motion imposes increased stress on adjacent joints, potentially leading to degenerative lesions in the midfoot and forefoot and arthritis in adjacent joints [17,18]. Concerning union rate, open AA utilizing internal fixation methods, such as the tripod screw technique, has been documented to range between 80% and 100%. Similarly, intramedullary nail tibiotalocalcaneal arthrodesis demonstrates comparable fusion rates, with reported AA rates between 89% and 100%, though slightly lower success rates are observed when concomitant subtalar fusion is performed [19,20]. TAA was developed as an alternative to AA and has been increasingly adopted for the treatment of end-stage ankle arthritis [17,19]. With advancements in surgical techniques and implant design, the clinical outcomes of TAA have shown corresponding improvements. Comparative literature, including a randomized controlled trial, indicates that TAA is associated with a higher incidence of wound-healing complications and nerve injuries, whereas AA carries a greater risk of thromboembolism and nonunion, with a symptomatic nonunion rate of 7%. Additionally, TAA may have a higher revision rate due to instability, technical errors, cyst formation [21], and implant loosening [22]. Both TAA and AA are effective treatments for end-stage ankle arthritis, but the choice of procedure must be tailored to the individual patient’s needs and circumstances [23,24,25]. This study utilizes an extensive dataset of 27,595 patients to provide valuable insights into the impact of TAA and AA on patient care and healthcare resource utilization. Our primary objective is to contribute to the ongoing discourse on the efficacy of these surgical approaches by enhancing the understanding of their practical implications, benefits, and disadvantages. In addition, our secondary objective is to compare the incidence of intraoperative and postoperative complications between the two groups. Through this investigation, we aim to offer meaningful insights to guide future research and clinical practice, ultimately optimizing patient-centered care and healthcare resource allocation.

## 2. Methods

### 2.1. Data Source

This study utilized the National Inpatient Sample (NIS), a comprehensive and publicly available database developed by the Agency for Healthcare Research and Quality (AHRQ) as part of the Healthcare Cost and Utilization Project (HCUP). The NIS is the largest all-payer inpatient healthcare database in the United States, capturing approximately 20% of all hospital discharges from HCUP-affiliated institutions. This dataset, comprising approximately 7 million unweighted inpatient admissions annually, enables the derivation of national estimates through the application of discharge sample weights provided by the NIS. The study dataset spans 1 January 2016 to 31 December 2019, representing the most recent available data at the time of analysis. Each individual dataset entry, hereafter referred to as a “case”, corresponds to a discharge-weighted group of five patients, in accordance with the weighting methodology employed by the NIS. It is important to note that the database used in this study captures only inpatient information and does not include outpatient follow-up data. As a result, outcomes that occur after discharge—such as long-term complications, functional outcomes, or delayed reoperations—could not be assessed. Ethical approval was obtained from the relevant institutional review board (IRB), with a waiver of informed consent granted due to the de-identified and anonymized nature of the NIS dataset.

### 2.2. Cohort Definition and Selection Criteria

The NIS database was systematically queried for the years 2016–2019 to identify adult patients (aged >18 years) who underwent elective TAA or AA during the study period. Given the discharge-weighted sampling methodology of the NIS, each dataset entry, hereafter referred to as a “case,” represents a group of five weighted patients. The initial cohort included 4834 cases of TAA, corresponding to an estimated 24,170 patients, and 2465 cases of AA, representing a weighted total of 12,325 patients. To enhance data homogeneity and mitigate potential confounding due to variations in underlying pathology, the study was restricted to patients diagnosed with primary osteoarthritis. This refinement yielded a final analytical cohort of 20,765 patients undergoing TAA and 6830 patients undergoing AA. Admissions classified as non-elective were systematically excluded to ensure a more controlled study population.

### 2.3. Statistical Analysis

All statistical analyses were conducted using SPSS 26 and MATLAB 2024 to compare TAA and AA, employing cross-tabulations and independent sample *t*-tests for categorical and continuous variables, respectively, with a two-tailed significance threshold of *p* < 0.05. To mitigate selection bias and confounding inherent in observational studies, propensity score matching (PSM) was implemented to create statistically comparable cohorts by matching patients undergoing TAA and AA on key demographic, hospital-related, and clinical characteristics, thereby simulating the conditions of a randomized controlled trial (RCT) and enhancing the validity of causal inferences. The propensity score was estimated through a logistic regression model incorporating 34 covariates spanning three major domains: hospital characteristics, including hospital size, type (urban vs. rural), teaching status, geographic region, and total annual discharges; demographic and socioeconomic factors, including patient location (urban vs. rural classification), median household income quartile, race, age, and primary payer status (Medicare, Medicaid, private insurance, self-pay, or other); and comorbidities and preoperative conditions, accounting for 24 preoperative comorbidities, including hypertension, dyslipidemia, obstructive sleep apnea, chronic anemia, alcohol abuse, osteoporosis, neurodegenerative diseases (Parkinson’s disease, Alzheimer’s disease, and dementia), chronic kidney disease, congestive heart failure, chronic lung disease, diabetes mellitus, inflammatory bowel disease, liver disease, obesity, fibromyalgia, thyroid disorders, history of myocardial infarction, peripheral vascular disease, history of cerebrovascular accident, any neoplasm, and neoplasms of lymphoid and hematopoietic tissue. Following PSM, the refined dataset included 6830 matched cases per group, ensuring that both cohorts had comparable baseline characteristics, with matching criteria encompassing hospital size, patient location (urban–rural classification), median household income, hospital geographic region, preoperative comorbidities, and total annual hospital discharges recorded in the NIS dataset. This methodological approach enhances internal validity, reduces the influence of confounding variables, and strengthens the robustness of comparative assessments between TAA and AA.

### 2.4. Ethical Consideration

The study received exempt status from the Institutional Review Board (IRB) due to the de-identified nature of the National Inpatient Sample (NIS) dataset, ensuring full compliance with ethical standards for human subject research. Artificial intelligence (AI) tools were employed exclusively for enhancing linguistic clarity, grammatical accuracy, and stylistic refinement of the manuscript. These tools were not utilized for data analysis, statistical interpretation, or content generation, thereby preserving the integrity of the research methodology and findings.

## 3. Results

Our analysis of the Nationwide Inpatient Sample from 1 January 2016 to 31 December 2019 investigated TAA relative to AA procedures. As shown in Table 1, osteoarthritis is the primary reason for TAA and AA procedures. Due to the potential for coding errors by surgeons in the U.S. healthcare system—where post-traumatic cases are often inaccurately recorded as primary osteoarthritis—we combined both post-traumatic and primary osteoarthritis cases under a unified “ankle osteoarthritis” category to minimize misclassification bias in our analysis. Thus, it accounted for 91.2% of TAA and 59.9% of AA surgeries. Other etiologies include fractures, complications of internal orthopedic prosthetics, diabetes, leg deformity, Charcot joint, osteonecrosis, osteomyelitis, rheumatoid arthritis, and orthopedic aftercare.

**Figure 1:**
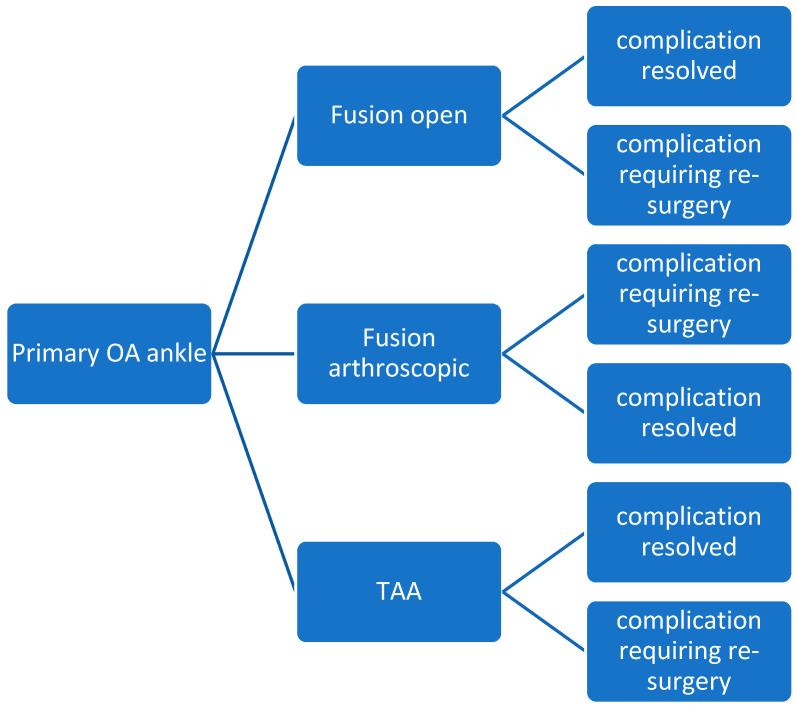
Surgical treatment methods of ankle osteoarthritis.

From this point, we included only patients with primary osteoarthritis to maintain data homogeneity. This sub-group analysis allowed us to focus on a more comparable patient population and mitigate potential biases arising from different etiologies. Figure 1 presents the treatment options of ankle osteoarthritis.

**Figure 2:**
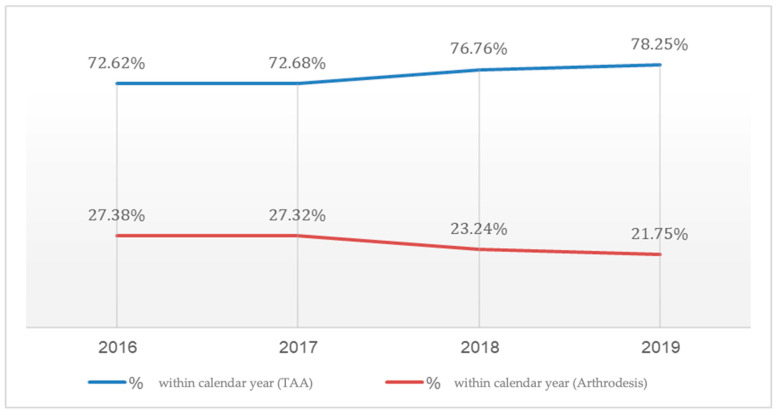
Percentage of total ankle arthroplasty and ankle arthrodesis procedures within each calendar year (2016–2019).

Examining the proportion of TAA procedures among all ankle arthroplasties, we observed a statistically significant increase (*p* < 0.0001) in the prevalence of TAA over the study period. This trend manifested as a sharp rise in the percentage of TAA procedures from 73% in 2016 to 78% in 2019, while the utilization of AA decreased from 27% in 2016 to 22% in 2019, as shown in Figure 2.

As shown in Table 2, 20,765 patients underwent TAA, whereas 6830 patients underwent AA. The average age for patients undergoing TAA was significantly higher (65.61 years) than those undergoing AA (59.66 years). When examining payer characteristics, Medicare was the most common payer for both procedures, but significantly more so for TAA (59.3%) compared to AA (47.8%) (*p* < 0.0001). Racial demographics showed that Caucasian patients were more likely to undergo TAA (90.1%) compared to the AA group (83.5%) (*p* < 0.0001).

Table 3 examines the differences in comorbidities between TAA and AA. Patients undergoing AA had higher rates of type 2 diabetes (24.9% vs. 13.8%), chronic lung disease (9.5% vs. 4.5%), obesity (33.2% vs. 22.2%), and mental disorders (36.6% vs. 26.4%) (all *p* < 0.0001). Additionally, renal disease, CHF, and obstructive sleep apnea were more common in the AA group compared to the TAA group.

Using propensity score matching, the analysis offers comprehensive insights into demographics, payer information, and the prevalence of various medical conditions, illustrating parameters such as average age, gender distribution, payer type, and an array of diagnoses within both the TAA and AA groups. The propensity score-matched analysis data, as presented in Table 4, reveal no significant differences across most examined parameters except in payer, underscoring the homogeneity of the two patient cohorts and demonstrating the efficacy and reliability of the applied propensity score-matched analysis.

The comparative results regarding hospitalization duration, post-procedural outcomes, and overall analysis between the TAA and AA groups are detailed in Table 5. A notably higher mortality rate was observed in the AA group (*p* = 0.002). Furthermore, significant differences were identified in both the average length of stay and the mean total charges, with the TAA group demonstrating more favorable outcomes (*p* < 0.0001). Postoperative complication rates are shown in Table 6. AA was associated with higher rates of blood loss anemia, acute kidney injury, and heart failure. Additionally, surgical wound complications, blood transfusion rate, and pneumonia were more prevalent in the AA group. Acute coronary artery disease, pulmonary edema, venous thromboembolism, and pulmonary embolism did not show significant differences between the groups.

## 4. Discussion

The current study, utilizing a large cohort of patients and minimizing selection bias through propensity score matching, revealed significant differences between TAA and AA. Our analysis demonstrated a statistically significant increase in the prevalence of TAA procedures over the study period, with TAA utilization rising from 73% in 2016 to 78% in 2019, while AA decreased from 27% to 22%, which has been reported in various centers in the world. [26,27] In addition, while TAA is associated with higher initial costs, it provides better outcomes in terms of reduced in-hospital mortality, shorter hospital stays, and lower postoperative complication rates compared to AA. These findings suggest that, despite AA traditionally being regarded as the gold standard option for end-stage ankle osteoarthritis, the superior outcomes with TAA in this study call that assumption into question. The data suggest that it may be appropriate to reconsider the role of AA as the preferred surgical option. While not definitive, these data prompt further discussion and exploration of TAA as a viable and potentially preferable alternative, especially given its advantages despite slightly higher costs.

Patients undergoing AA exhibited higher incidences of comorbidities, including type 2 diabetes, chronic lung disease, obesity, and mental disorders (all *p* < 0.0001). However, it is crucial to note that the groups cannot be directly compared due to differing indications for each procedure, highlighting the potential for selection bias. A comprehensive study revealed contradictory findings, indicating a higher Elixhauser Comorbidity Index in the TAA group, primarily due to arrhythmias, congestive heart failure, diabetes mellitus, electrolyte/fluid disorders, and iron deficiency anemia [18]. Additionally, a large retrospective study from Japan reported that patients undergoing TAA were generally older, more likely to be female, and had a higher prevalence of rheumatoid arthritis compared to those undergoing AA [28].

Moreover, AA patients faced higher in-hospital mortality rates and longer hospital stays. These findings align with a previous study on the same database evaluating trends in TAA [29]. In terms of mortality, our results corroborate the existing literature [27,28]. Importantly, our analysis indicates that the choice to perform AA or TAA is significantly influenced by patient characteristics, making propensity score matching essential for obtaining comparable groups. Our results are consistent with other studies demonstrating a significant reduction in in-hospital length of stay (LOS) over time for TAA patients compared to AA patients, with some TAA patients even being discharged on the same day [30]. Additionally, another study found that LOS was associated with age, indicating that patients older than 70 years have an increased risk for prolonged hospital stays and discharge to non-home settings [31]. As mentioned previously, AA patients generally present with more comorbidities and face higher mortality and longer hospital stays compared to TAA patients, who tend to be older and more likely to have rheumatoid arthritis. These differences underscore the importance of addressing selection bias in our analysis and the need for propensity score matching to ensure meaningful comparisons between these groups.

Medicare was the predominant payer for both TAA and AA, with a higher prevalence of Medicare coverage in TAA cases. The hospitalization charges for TAA were significantly higher than for AA, amounting to $91,358 compared to $79,308. A literature review indicates that TAA is consistently associated with higher costs. For instance, a study utilizing the National Inpatient Sample demonstrated a substantial increase in TAA costs, which rose from $40,203 in 2005 to $86,208 by the end of 2013, with a continuing upward trend thereafter [28]. It is important to note that cost structures can vary significantly across different countries; thus, these findings may not be universally applicable. However, given that our analysis is based on real-life data for the investigated patient population, it holds definitive value in understanding the economic implications of TAA and AA within this specific context.

Postoperative complications were higher among the AA group, with significantly elevated rates of blood loss, anemia, acute kidney injury, and heart failure (all *p* < 0.0001). Risk ratio analysis further highlighted the significantly increased risks of infection, blood transfusion, heart failure, and in-hospital mortality in the AA group, indicating a greater overall complication burden compared to TAA. However, it is important to recognize that these findings are based on unadjusted comparisons. Using propensity score matching, which minimizes selection bias by aligning groups based on similar characteristics, we found a marked reduction in these differences. The propensity score-matched analysis revealed no significant differences in most examined parameters except payer. Our analysis indicated significantly lower in-hospital mortality rates and shorter length of stay in the TAA group.

The literature on postoperative complications remains contradictory; for instance, one study indicated a higher need for blood transfusions in AA patients, while others have correlated lower hospital volume and shorter anesthesia duration with higher rates of adverse events following TAA [19]. Furthermore, a review of a large meta-analysis showed that the relative risk of complications improved with advancements in implant design, addressing concerns such as nerve injury, fractures, wound complications, and radiological abnormalities like heterotopic bone formation and aseptic loosening [32].

While our study focuses on early outcomes and complications between TAA and AA, it is important to acknowledge advancements in prosthetic designs and surgical techniques that have improved the long-term success and survival rates of TAA. Modern ankle replacements now demonstrate longevity comparable to hip and knee replacements [33]. However, these long-term benefits, along with the associated higher initial hospitalization costs and the need for more frequent follow-up care to monitor for complications such as implant loosening or wear, are outside the scope of this paper. Future research could investigate these aspects to provide a more comprehensive understanding of the long-term efficacy and economic implications of TAA compared to AA, particularly in patients seeking to maintain an active lifestyle.

Concerning gait biomechanism, patients undergoing TAA typically experience greater postoperative motion in the sagittal plane—namely dorsiflexion and plantarflexion—compared to those treated with AA. This improved mobility often translates into more favorable gait patterns, including increased walking speed, longer stride length, and higher cadence. However, plantarflexion remains limited compared to healthy controls [34,35,36,37]. In contrast, patients who undergo AA generally have reduced ankle motion and tend to compensate with increased hip range of motion. Their gait is marked by diminished ankle moments and power, along with higher transient forces at heel strike. Despite these limitations, many AA patients still achieve functional gains, including improved walking speed and enhanced ankle moment, following surgery [34,35,38].

Looking into patient satisfaction, patients who undergo TAA often experience meaningful relief from pain and a notable improvement in daily function, with many reporting higher levels of satisfaction compared to those treated with AA. Studies show that TAA is associated with better functional outcomes and higher scores on patient-reported outcome measures (PROMs), including the Foot and Ankle Ability Measure (FAAM) and Short Form-36 (SF-36) [39,40,41]. While AA also provides substantial pain relief and helps patients regain function, satisfaction rates tend to be slightly lower. PROMs such as the American Orthopaedic Foot and Ankle Society (AOFAS) scores do show clear postoperative improvement, but gait irregularities and compensatory movement patterns often persist [12,39,42]. In summary, both procedures offer important benefits for patients with end-stage ankle arthritis, but TAA may provide a more natural gait and a higher overall level of patient satisfaction in many cases.

While the NIS offers valuable insights into inpatient hospital stays, it comes with certain limitations that should be acknowledged. Because the database does not include outpatient or long-term follow-up data, we were unable to assess health outcomes beyond the initial hospital stay. Additionally, it lacks detailed clinical information such as laboratory values or comprehensive medical histories, which limits the depth of clinical interpretation. Another important limitation is the potential for coding errors or inconsistencies in data entry, which may impact the accuracy of our findings. One limitation worth noting is the issue of diagnostic coding accuracy in large administrative databases. While post-traumatic osteoarthritis is known to be the most common cause of ankle arthritis, our dataset showed an unexpectedly high number of cases listed as primary osteoarthritis. This likely reflects a common challenge in the U.S. healthcare system, where the reason for surgery is sometimes miscoded. In practice, surgeons may label a case as primary osteoarthritis even when there is a clear history of trauma, often due to time constraints or the lack of standardized coding guidelines. As a result, primary osteoarthritis may appear overrepresented in the data, not because it is truly more common, but because of how it is recorded. We believe this explains the imbalance seen in our study.

Cost analyses based on hospital charges may also not fully reflect the true costs of care, adding another layer of complexity when interpreting economic outcomes. These limitations highlight the importance of interpreting our results with caution and suggest that our conclusions should be seen as informative but not definitive.

This study also has several notable strengths. To the best of our knowledge, it is the first to compare TAA and AA using the NIS, offering a broad, nationally representative overview of current clinical practices. By examining data from multiple years, we were able to track changing trends and analyze outcomes in a much larger and more diverse patient population than previous studies. We also took into account differences in socioeconomic factors and hospital characteristics, allowing us to explore how these variables impact healthcare costs and resource use. Importantly, this is the first study to make this comparison using ICD-10 coding, which aligns with how procedures are currently recorded and reimbursed. This adds practical relevance to our findings and enhances comparability with future research using similar methodology.

Our methodology, which utilized extensive ICD-10 codes from a large dataset, provides a macro-level perspective rooted in real-life clinical practice. While this approach lacks detailed patient-level data, the inclusion of over 20,000 cases enhances the statistical power of our findings. Unlike randomized controlled trials (RCTs), where surgeries are often performed by highly specialized professionals, our registry-based study reflects the realities of everyday clinical practice, offering valuable insights into the outcomes in broader, more typical patient populations. Although registry studies have limitations, their ability to mirror real-world scenarios is perhaps even more significant in understanding the true effectiveness of these procedures.

It is imperative that future studies delve deeper into the efficacy of TAA, an area not fully explored in our current research. Understanding the suitability and benefits of TAA in more complex cases is a pressing need for enhancing clinical decision-making and patient care.

## 5. Conclusions

In conclusion, our analysis revealed that primary osteoarthritis was the leading cause of TAA and AA procedures. There was a significant increase in TAA prevalence over the study period, while AA procedures decreased. AA patients exhibited higher rates of comorbidities. In addition, AA was associated with higher in-hospital mortality, extended hospital stays, and postoperative complications compared to TAA. Risk ratio analysis underscored significantly higher risks for infection, blood transfusion, heart failure, and in-hospital mortality in the AA group, highlighting the increased complication and mortality risks associated with AA compared to TAA.

## Figures and Tables

**Table 1:** Distribution of etiologies for total ankle arthroplasty and ankle arthrodesis.

Etiology for Surgery	Total Ankle Arthroplasty	Ankle Arthrodesis	Significance
Osteoarthritis	91.20%	59.90%	*p* < 0.0001
Fracture	0.20%	13.50%	
Complications of internal orthopedic prosthetic	6.70%	8.20%	
Diabetes	0.00%	6.40%	
Leg Deformity	0.40%	5.40%	*p* < 0.0001
Charcot joint	0.00%	2.40%	
Osteonecrosis	0.20%	1.80%	
Osteomyelitis	0.00%	1.30%	
Rheumatoid arthritis	0.90%	0.60%	
Orthopedic aftercare	0.30%	0.50%	

**Table 2:** Demographic and payer characteristics for total ankle arthroplasty and ankle arthrodesis.

Parameter	Total Ankle Arthroplasty (n = 20,765)	Ankle Arthrodesis (n = 6830)	Significance
Total surgeries (count)	20765	6830	-
Average age	65.61	59.66	*p* < 0.0001
Female (%)	44.9%	46.0%	*p* = 0.137
Payer—Medicare (%)	59.3%	47.8%	*p* < 0.0001
Payer—Medicaid (%)	3.7%	11.3%	
Payer—Private (%)	33.1%	33.7%	
Payer—Other (including self-pay) (%)	4.00%	7.30%	
Race—White (%)	90.1%	83.5%	*p* < 0.0001
Race—Black (%)	2.9%	6.2%	
Race—Hispanic (%)	3.5%	6.3%	
Race—Asian or Pacific Islander (%)	0.9%	1.2%	
Race—Native American (%)	0.3%	0.8%	
Race—Other (%)	2.3%	2.0%	

**Table 3:** Comorbidities for total ankle arthroplasty and ankle arthrodesis.

	Total Ankle Arthroplasty (n = 20,765)	Ankle Arthrodesis (n = 6830)	Significance
Hypertension diagnosis (%)	53.4	50.9	*p* < 0.0001
Dyslipidemia diagnosis (%)	39.5	37.8	*p* = 0.011
Chronic anemia (%)	2.8	4.5	*p* < 0.0001
Osteoporosis (%)	3.6	2.3	*p* < 0.0001
Alcohol abuse (%)	1.1	2.3	*p* < 0.0001
Type 2 diabetes (%)	13.8	24.9	*p* < 0.0001
Renal disease (%)	5.4	9.4	*p* < 0.0001
CHF (%)	0.8	2.1	*p* < 0.0001
Chronic lung disease (%)	4.5	9.5	*p* < 0.0001
Obesity (%)	22.2	33.2	*p* < 0.0001
IBD	0.3	0.2	*p* = 0.34
Coagulation defects	0.6	1.3	*p* < 0.0001
Connective tissue disorder	0.1	0.3	*p* = 0.002
Mental disorder	26.4	36.6	*p* < 0.0001
Parkinson’s disease	0.7	0.7	*p* = 0.938
Obstructive sleep apnea	11.5	15.8	*p* < 0.0001

**Table 4:** Demographic and payer characteristics for total ankle arthroplasty and ankle arthrodesis after propensity score matching.

Parameter	Total Ankle Arthroplasty (n = 6830)	Ankle Arthrodesis (n = 6830)	Significance
Total surgeries (count)	6830	6830	-
Average age	60.07	59.66	*p* = 0.06
Female (%)	47.2%	46.0%	*p* = 0.145
Payer—Medicare (%)	47.0%	47.8%	***p* < 0.02**
Payer—Medicaid (%)	8.9%	11.3%	
Payer—Private (%)	39.5%	33.7%	
Payer—Other (including self-pay) (%)	4.60%	7.30%	
Race—White (%)	83.2%	83.5%	*p* = 0.08
Race—Black (%)	6.5%	6.2%	
Race—Hispanic (%)	6.3%	6.3%	
Race—Asian or Pacific Islander (%)	1.0%	1.2%	
Race—Native American (%)	0.7%	0.8%	
Race—Other (%)	2.3%	2.0%	
Hypertension diagnosis (%)	51.1%	50.90%	*p* = 0.13
Dyslipidemia diagnosis (%)	36.40%	37.80%	*p* = 0.09
Chronic anemia (%)	4.2	4.5	*p* = 0.40
Osteoporosis (%)	2.5	2.3	*p* = 0.40
Alcohol abuse (%)	2.3	2.3	*p* = 0.52
Type 2 diabetes (%)	25.3	24.9	*p* = 0.07
Renal disease (%)	9.9	9.4	*p* = 0.31
CHF (%)	2.4	2.1	*p* = 0.14
Chronic lung disease (%)	8.6	9.5	*p* = 0.07
Obesity (%)	33.5	33.2	*p* = 0.26
IBD	0.3	0.2	*p* = 0.40
Coagulation defects	1.2	1.3	*p* = 0.18
Connective tissue disorder	0.3	0.3	*p* = 1
Mental disorder	35.8	36.6	*p* = 0.08
Parkinson’s disease	0.5	0.7	*p* = 0.26
Obstructive sleep apnea	15.2	15.8	*p* = 0.29

**Table 5:** Comparison hospitalization outcomes after propensity score matching.

	Total Ankle Arthroplasty (n = 6830)	Ankle Arthrodesis (n = 6830)	Significance
Died during hospitalization	0.00%	0.15%	*p* = 0.002
Length of stay (mean in days)	1.77	2.49	*p* < 0.0001
Total charges (mean in $)	89,808	79,308	*p* < 0.0001

**Table 6:** Comparison of postoperative complications for total ankle arthroplasty and ankle arthrodesis in a propensity score-matched cohort.

	Total Ankle Arthroplasty (n = 6830)	Ankle Arthrodesis (n = 6830)	Significance
Blood loss anemia	3.8%	7.91%	*p* < 0.0001
Acute kidney injury	0.7%	2.86%	*p* < 0.0001
Heart failure	0.00%	0.22%	*p* < 0.0001
Acute coronary artery disease	0.15%	0.15%	*p* = 1
Pulmonary edema	0.14%	0.07%	*p* = 0.196
Venous thromboembolism	0.07%	0.14%	*p* = 0.196
Pulmonary embolism	0.07%	0.07%	*p* = 1
Pneumonia	0.00%	0.37%	*p* < 0.0001
Surgical wound complication	0.00%	0.51%	*p* < 0.0001
Blood transfusion	0.20%	1.90%	*p* < 0.0001

## Data Availability

Restrictions apply to the availability of these data. Data were obtained from HCUP and are available [https://hcup-us.ahrq.gov/] with the permission of HCUP.

## List of Abbreviations

AA	Ankle arthrodesis
HCUP	Healthcare Cost and Utilization Project
ICD-10	International Classification of Diseases, 10th revision
LOS	Length of stay
NIS	Nationwide Inpatient Sample
SPSS	Statistical Package for the Social Sciences
TAA	Total ankle arthroplasty
